# Combined parecoxib and I.V. paracetamol provides additional analgesic effect with better postoperative satisfaction in patients undergoing anterior cruciate ligament reconstruction

**DOI:** 10.4103/1658-354X.76510

**Published:** 2011

**Authors:** Zeinab Ahmed Elseify, Salwa Omar El-Khattab, Ahmed Metwally Khattab, Eman Mohammed Atta, Layal Fares Ajjoub

**Affiliations:** 1 *AL-Ahli Hospital, Doha, Qatar*; 2 *AinShams University, Cairo, Egypt*; 3 *Doha Clinic Hospital, Doha, Qatar*; 4 *Elzagazig University, Elzagazig, Egypt*

**Keywords:** *Intravenous paracetamol*, *parecoxib*, *postoperative analgesia*

## Abstract

**Background:**

Adequacy of postoperative analgesia is one of the most important factors that determine early hospital discharge and patients’ ability to resume their normal activities postoperatively. The optimal non-opioid analgesic technique for postoperative pain management would reduce pain and enhance patient satisfaction, and it also facilitates earlier mobilization and rehabilitation by reducing pain-related complications after surgery. The aim of this study was to evaluate the analgesic efficacy of intravenous paracetamol and parecoxib when used alone, or in combination.

**Methods:**

Sixty American Society of Anesthesiology (ASA) physical status I and II adult patients who were scheduled for anterior cruciate ligament reconstruction were included in this study. Patients were allocated into three groups: group I patients received 1g intravenous paracetamol after induction and another 1 g 4 h later, group II received 40 mg parecoxib after induction, while group III received combination of both drugs (paracetamol 1 g and parecoxib 40 mg). Pain during rest and mobility was assessed in the immediate postoperative period, 2 h and 8 h successively using visual analog scale (VAS). Patient satisfaction was rated according to satisfaction score.

**Results:**

Total morphine requirements were lower in group III patients (6.9±2.7 mg) in comparison to group I patients (12.6±3.6 mg) or group II patients (9.8±2.8 mg). The least VAS scores were recorded during knee movement (3.8±1.1) in group III patients compared to group I (6.0±1.8) and group II patients (4.8±1.9). Eight hours postoperatively, group III patients were more satisfied regarding the postoperative pain management.

**Conclusion:**

Combination of intravenous paracetamol and parecoxib provided better analgesia and higher patient satisfaction than each drug when used separately.

## INTRODUCTION

Despite the availability of various therapeutic approaches to pain management and an improved understanding of pain pathophysiology, acute postoperative pain continues to be undertreated or treated ineffectively. Proper pain management is essential for the early recovery and rehabilitation especially after knee surgery. Recent evidence suggests that this goal can be best achieved by using a combination of pre-emptive techniques involving both centrally and peripherally acting analgesic drugs and devices.[1] Opioids are effective analgesics, but their usefulness is limited by side effects, such as nausea and vomiting, somnolence, constipation, and respiratory depression.[2] Non- opioids (e.g., paracetamol, non-steroidal anti-inflammatory drugs NSAID, and local anesthetics) have opioid sparing effects.[3]

Cyclo-oxygenase (COX)-2 selective inhibitor drugs (coxibs) are thought to have beneficial effects on inflammation and pain with concomitant preservation of homeostatic function and reduction in the incidence of side effects as compared with nonselective NSAIDs. Parenteral formulations allow for intra-operative administration and may overcome the problem of bioavailability encountered with oral formulations within the perioperative setting. Parecoxib is the first injectable COX-2 selective inhibitor indicated for the treatment of acute postoperative pain.[4] It is an inactive pro-drug that undergoes rapid amidehydrolysis *in vivo* to the pharmacologically active, highly specific inhibitor of cyclooxygenase-2 (COX-2) enzyme, valdecoxib.[5,6] Parecoxib and valdecoxib were not found to increase the risk of cardiovascular adverse events after non-cardiac surgery.[7]

Paracetamol (acetaminophen) is an effective and safe analgesic used worldwide to relieve mild to moderate pain in conditions such as headache, toothache, and arthritis.[8] Acetaminophen and NSAIDs probably have different sites of action; their combined use may have additive or synergistic effect. The objective of this study was to compare the analgesic effect of parecoxib and intravenous paracetamol given separately or together on the early postoperative pain and to evaluate patients’ satisfaction in patients undergoing ACL under general anesthesia.

## METHODS

After obtaining institutional approval (Doha Clinic Hospital-Doha-Qatar) and informed written consent, a prospective, randomized, double blind study was conducted from July 2007 through August 2008. Sixty ASA physical status I and II patients, aged between 18 and 45 years scheduled for elective ACL reconstruction surgery were participated in this study. Exclusion criteria were pregnancy, breast-feeding women, history of drug abuse, or allergy to any of the study medications, intake of narcotic analgesics, NSAIDs, or paracetamol within 24 h before the study. All patients were premedicated with 7.5 mg midazolam tablet 1 h before surgery. Patients enrolled in the study were randomly allocated by computer-generated random numbers to be divided into three groups: group I (paracetamol group) 20 patients, group II patients (parecoxib group) 19 patients, and group III (paracetamol--parecoxib group) 21 patients.

During the preoperative visit, each enrolled patient was asked to choose a sealed envelope with his code number inside. The name, file number, and body weight were recorded on the chosen sealed envelope. The envelopes were opened before the start of anesthesia. Anesthesia induction was performed with propofol (Diprivan^®^ 1% Astra-Zeneca, Madrid) 2-3 mg/kg, Fentanyl 2 μg/kg induction dose, increments of Fentanyl were added according to the intraoperative requirements, cisatracurium (Nimbex^®^ -Glaxo Smith Kline, S.A. Spain) 0.15 mg/kg. All patients were mechanically ventilated after insertion of laryngeal mask (LMA-Classic^™^) with 40:60 oxygen and nitrous oxide. Anesthesia was maintained with Sevoflurane (Abbott) 1.50±0.50 Vol%. Group I received 1 g IV Paracetamol (Perfalgan^®^ 100 ml vial UPSA France) after induction and 1 g 4 h later, group II patients received 40 mg IV Parecoxib (Dynastat^™^ PHARMACIA) after induction, and group III patients received both parecoxib and paracetamol at induction and 1 g paracetamol after 4 h.

**Figure 1:**
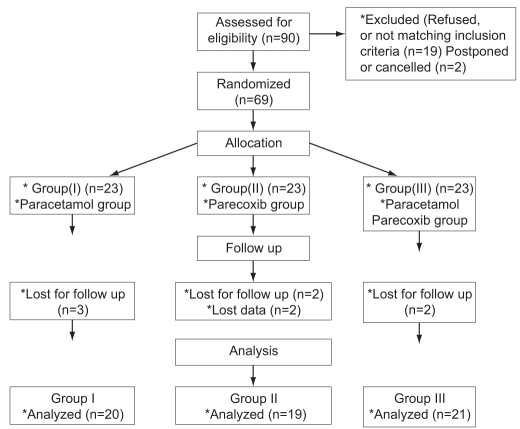
Flowchart of patients through the trial

Paracetamol was administered by slow infusion over 15 min, whereas parecoxib was injected as a rapid bolus. Each patient in groups I and II received the proposed drug and the placebo of the other drug. Operations were carried out by the same surgeon, who was blinded to the drugs administered. Local anesthetics were avoided in all patients under the study. At the end of the procedure, residual paralysis was antagonized with neostigmine and atropine if needed. After laryngeal mask removal, the patients were transferred to the post-anesthesia care unit (PACU). Pain intensity at rest and during active knee movement was assessed immediately upon full recovery in the PACU using a 10 cm visual analogue scale (VAS) {0 = no pain → 10 = worst imaginable pain}. IV morphine boluses (3 mg) were given and possibly repeated every 15 min with a maximum dose of 12 mg, until VAS 3 or less. Patients would not be discharged to the ward unless being awake and oriented, able to move all extremities on command, hemodynamically stable, respiratory stable (able to breathe deeply), oxygen saturation >90% on room air, no or mild discomfort, and with no or mild nausea but no vomiting. After leaving the PACU, pain was re-assessed during rest and active movement after 2 h and 8 h using VAS. Patient’s satisfaction with postoperative pain management was assessed at the 8th hour postoperatively with a 4-point rating scale (poor= 0, fair= 1, good= 2, and excellent= 3). IV morphine 3 mg was given upon request in cases of persistent pain until VAS 3 or less. Total doses of morphine given in the PACU and in the ward until 8 h were calculated. Flow chart of patients through the trial is shown in Figure 1.

Analysis of data was done by IBM computer using SPSS (statistical program for social science). The paired student t-test was used to compare quantitative data in the same group. One-way ANOVA test (analysis of variance) was used to compare more than two groups as regards quantitative variables. *P* value <0.05 was considered statistically significant.

## RESULTS

Sixty patients (20 in the paracetamol group, 19 in the parecoxib group and 21 in the paracetamol--parecoxib group) were included from July 2007 through August 2008. Demographic data, procedure duration, intraoperative fentanyl consumption and duration of stay in PACU are presented in Table 1.

**Table 1:** Patient’s demographic and operative data

	Group I (Paracetamol group)	Group II (Parecoxib group)	Group III (Combined group)
N	20	19	21
Age (year)	33.15±7.1	334±6.8	31.6±8.5
Body weight (Kg)	74 95±94	75.26±8.9	76.47±9.8
Sex (male/female) (N)	17/3	15/4	18/3
Duration of surgery (min)	57.8±6.5	58.5±6	55.3±7.2
Fentanyl consumption (μg)	162±19.49	160.5±24	158.3±25.4
Stay in (PACU) (min)	31.35 ±439	23.48±3.8	17.23±2.34*

* Significant (*P* >0.05), PACU = Post-anesthesia care unit

There were no inter-group differences in patients’ demographics, duration of surgery and intraoperative fentanyl consumption. There were statistically significant differences between group II (23.48±3.8 min) and group III (17.23±2.34 min) and high statistically significant difference between group I (31.35±4.39 min) and group III (17.23±2.34 min) with no statistically significant difference between group I (31.35±4.39 min) and group II (23.48±3.8 min) as regards the time of stay in PACU. Doses of morphine early postoperatively and total doses were lowest among group III patients (2.3±1.9 and 6.9±2.7 mg) which is then followed by group II (4.4±2.7 and 9.8±2.8mg), and there was a high statistically significant difference between group III versus group I and group II and between group I, and group II by ANOVA test (*P*<0.01) as shown in Table 2.

**Table 2:** Changes in morphine doses within each group and difference between the studied groups at different intervals

The studied groups	Early postoperative	After 2 h	After 8 h	Total morphine
Group I N=20	7.05±2.6(b,c)	2.7±19	5.6±2.1	12.6±3.6 (b,c)
*P*		<0.001**	>0.05	
Group II N=19	4.4±2.7(a,c)	2.5±1.8	54±1.6	9.8±2.8 (a,c)
*P*		<0.05*	>0.05	
Group III	2.3±1.9(a,b)	2.1±1.9	4.6±2.03	6.9±2.7 (a,b)
N = 21				
*P*		0. 2 4 >0.05	3.5 <0.01**	
Significance test between the groups (ANOVA test)	F=22	F=0.45	F=1.5	F=18
	*P*<0.001**	*P*>0.05	*P* >0.05	*P*<0.001**

* Significant test *P*<0.05

** Highly significant *P*<0.01, a-group I, b-group II, c-group III

In Table 3, VAS shows that during knee movement, group III patients had the lowest pain compared to the other two groups and also group II had less pain than group I with a high statistically significant difference between group III (3.8±1.1) versus both group I (6.0±1.8) and group II (4.8±1.9) and also a high statistically significant difference between I versus group II by ANOVA test (*P*<0.01). On the other hand, no statistically significant difference could be detected between the all groups as regard VAS during rest in the early postoperative period by the same test *P*>0.05.

**Table 3:** Changes in visual analogue scale within each group and differences between the studied groups during rest and movement at early postoperative period

The studied groups	Resting	Movement	t	*P*
Group I N=20	43±1.5	6.0±1.8(b,c)	3.3	<0.01**
Group II N = 19	4.2±2.2	4.8±1.9(a,c)	2.2	<0.05*
Group III N= 21	32±2	3.8±1.1(a,b)	1. 2	> 0. 05
Significance test between the groups (ANOVA test)	F=1.8	F=9.00		
	*P* >0.05	*P*<0.001**		

* Significant test *P*<0.05

** Highly significant *P*<0.01, a-group I, b-group II, c-group III

VAS values were higher during movements in comparison to rest within each group by the paired *t*-test (*P*<0.01), as shown in Table 4. After 8 h, no statistically significant difference could be detected between the all groups as regards VAS both during rest and movements.

**Table 4:** Changes in visual analogue scale at rest and during movement within each group and difference between the studied groups as regard visual analogue scale at rest and movement at 2 h and 8 h interval

The studied groups	Resting at 2 h postoperative	Resting 8 h postoperative	Movement 2 h postoperative	Movement 8 h postoperative
Group I N=20	3.1±1.7	2.3±13	45±1.9	33±1.3
*P*		> 0. 05	<0.001^**^	> 0. 05
Group II N = 19	2.3±1.7	2.3±1.2	4.3±1.7	33±1.2
*P*		> 0. 05	<0.001^**^	> 0. 05
Group III N= 21	2.1±1.5	1.7±1	3.8±1.7	2.6 ± 1
*P*		> 0. 05	<0.001^**^	> 0. 05
Significance test between the groups (ANOVA test)	F = 1.9, *P*>0.05	F = 2.1, *P*>0.05	F = 1.6, *P*>0.05	F = 2.00, *P*>0.05

Group III patients significantly considered pain management as good or excellent at the 8^th^ hour postoperatively with more satisfaction than the other two groups (*P*>0.05), [Table 5]. Although there was no statistically significant difference between groups I and II, still there was clinical evidence of higher patient satisfaction in group II than in group I.

**Table 5:** Patient’s satisfaction from postoperative pain management

Group I	Group II	Group I I I
1.7±0.55	2.0±0.41	2.5±0.39*

0= Bad, 1= Fair, 2= Good, 3= Excellent,

* Significant (*P*>0.05)

## DISCUSSION

Combined intravenous paracetamol and parecoxib exerted an additional analgesic effect in reducing pain intensity and rescue morphine consumption after ACL reconstruction surgery, particularly during the early postoperative period. Due to the longer duration of action of parecoxib (8-12 h), a second dose of paracetamol was repeated after 4 h to cover the time lag resulting from the shorter duration of paracetamol action (4-6 h). The higher morphine consumption during this period in patients who received either paracetamol or parecoxib alone could explain the statistically non-significant differences in both pain scores and the rescue analgesic doses during the following 8 h. Many studies have tested the analgesic efficacy of the combination of NSAIDs and paracetamol in its different forms: oral, rectal, or the relatively-old intravenous precursor propacetamol (Pro-Dafalgan^®^) in patients undergoing different surgical procedures. Unfortunately, there was no uniform conclusion.

Tijani *et al*. found that oral premedication with a combination of paracetamol 2 g and the COX_2_-inhibitor, celecoxib (200 mg) was effective in decreasing pain and improving patient satisfaction after otolaryngology surgery in comparison to either drug alone.[9]

On the other hand, combination of propacetamol with ketoprofen 50 mg has been shown to reduce pain scores at rest and on movement compared to ketoprofen alone after disk surgery, but there was no associated reduction in opioid requirement.[10] This was similar to the results of Siddik *et al*., who were unable to demonstrate the significant morphine-sparing effect of propacetamol when combined with rectal diclofenac in comparison to diclofenac alone after cesarean delivery in patients receiving patient-controlled analgesia using morphine.[11]

A majority of studies showed that NSAIDs seem to be superior to paracetamol in postoperative pain management, but the magnitude of this difference may depend upon the type of surgery performed.[12,13] Beaussier *et al*., found that single parenteral injection of parecoxib 40 mg compares favorably with two injections of propacetamol 2 g within the first 12 h after inguinal hernia repair in adult patients.[4]

Parecoxib being a selective COX2 inhibitor in usual doses is lacking COX1 enzyme inhibition. Recently, it has been suggested that COX1 enzyme plays an important role in spinal cord pain processing and sensitization after surgery.[14] In contrast, paracetamol inhibits COX1 enzyme activity in the central nervous system and might exert an analgesic effect via NMDA (N-methyl d-aspartate) receptors in the spinal cord.[15] Part of its effect is thought to be mediated via a central serotonergic mechanism as has been shown *in vivo*. However, 5-HT_3_ receptor antagonists did not directly antagonize paracetamol *in vitro*, and thus an indirect mechanism has been postulated.[16]

We concluded that intravenously administered paracetamol can provide an additional analgesic effect during the early post-operative period when combined with parecoxib intraoperatively in patients undergoing ACL reconstruction under general anesthesia in comparison to either drug alone.
